# Body ownership shapes self-orientation perception

**DOI:** 10.1038/s41598-018-34260-7

**Published:** 2018-10-30

**Authors:** Nora Preuss, B. Laufey Brynjarsdóttir, H. Henrik Ehrsson

**Affiliations:** Department of Neuroscience, Karolinska Institutet, Stockholm, Sweden

## Abstract

Self-orientation perception is a necessary ability for everyday life that heavily depends on visual and vestibular information. To perceive the orientation of oneself with respect to the external environment would seem to first require that one has a clear sense of one’s own body (‘sense of body ownership’). However, the experimental evidence for this is sparse. Therefore, the aim of the present study was to investigate how the sense of body ownership affects perceived self-orientation. We combined a self-orientation illusion – where the visual scene, i.e., a fully furnished room, was rotated slowly around the roll axis – with a full-body ownership illusion paradigm – where the ownership of a stranger’s body seen from the first-person perspective in the center of the scene was manipulated by synchronous (illusion) or asynchronous (control) visual-tactile stimulation. Participants were asked to judge the appearance of shaded disk stimuli (a shape-from-shading test), which are perceived as three-dimensional (3D) spheres; this perception depends on perceived self-orientation. Illusory body ownership influenced self-orientation as reported subjectively in questionnaires and as evident from the objective shape-from-shading test data. Thus, body ownership determines self-orientation perception, presumably by boosting the weighting of visual cues over the gravitational forces detected by the vestibular system.

## Introduction

How do you know what is up and what is down in the world? Maybe because the world looks upright, you might say. However, how do you know that the world is not tilted, and you too, to the same degree? Because you feel upright, so, therefore, the world must be upright, you might add. This example illustrates the intimate relationship between the orientation perception of the self and of the world. However, how is this relationship implemented in the human mind, more precisely? Self-orientation perception – the sense of what is up, down, left, right, and around us – is mainly determined through our vestibular sense – or our ‘sense of balance’. Our vestibular system is situated in the inner ear and is the main indicator of where our head is located in space. However, self-orientation perception requires not only information from our vestibular sense but also sensory information from our body. This information includes, for example, proprioceptive and tactile cues, indicating where our body and its different segments (i.e., limbs) are located in space relative to gravity but also to each other (for an overview, see^1^). Self-orientation perception is therefore a complex process that requires information from multiple sensory sources.

Over the last two decades, researchers have been increasingly interested in the question of how we come to experience our body as our own (the sense of body ownership)^2–4^. The sense of body ownership allows us to discriminate between that which is part of our own physical self and that which is part of the external world; this sense is fundamental for survival and constitutes a basic aspect of human self-consciousness^5–7^. The sense of body ownership arises from the dynamic integration of visual, tactile, vestibular, proprioceptive and other bodily signals into a coherent multisensory experience of one’s own body^6,8^. As mentioned above, the integration of multisensory information not only contributes to the sense of body ownership but also plays an important role in self-orientation perception. Thus far, however, the relationship between the sense of body ownership and self-orientation perception has remained unclear.

Previous studies investigating body ownership of an entire body used a perceptual illusion paradigm based on multisensory stimulation^9^. In this ‘full-body ownership’ illusion paradigm, participants see a mannequin’s body from the first-person perspective (1PP) while synchronous touches are applied to the participant’s real body and the mannequin’s virtual body. Simultaneous visuo-tactile stimulation leads to an illusory perception of ownership of the mannequin’s body; participants perceive the mannequin’s body as their own and sense the touches where they see them occur directly on the mannequin’s body^9–11^. Asynchronous visuo-tactile stimulation significantly reduces the illusion and serves as a good control while using otherwise equivalent conditions^9^.

Interestingly, perceived self-orientation can also be altered through a manipulation of visual cues inducing so-called ‘reorientation illusions’^12^. A reorientation illusion is characterized by a sudden change in self-orientation perception and is, for example, experienced by astronauts when gravitational information is absent and visual cues are ambiguous^13,14^. An ‘inversion illusion’ is a version of this illusion in which one feels that they are completely upside-down. Furthermore, reorientation illusions can also be induced by altering visual information under normal gravitational conditions^12^. In healthy participants, a continuous rotation of the visual environment (e.g., induced through virtual reality systems) provides such a strong visual motion cue that participants can experience a perception of self-motion and reorientation.

Although body ownership and self-orientation both require multisensory integration, a possible link between these two perceptual phenomena has not been addressed using the aforementioned paradigm. Interestingly, we know that perceived perspective can be influenced by visual, vestibular, and tactile signals^15–17^ in a paradigm where participants observe a body being stroked on the back from a third-person perspective while simultaneously receiving synchronous strokes on their own back^18^. However, this paradigm is based on a conflict between the visual perspective (third person perspective) and the visuo-tactile stimulation, which prohibits a coherent full-body ownership experience from emerging^11,19,20^. Moreover, neither this paradigm, nor the full-body ownership illusion described above, has been combined with a classic self-orientation paradigm to directly investigate interactions between self-orientation perception and the sense of bodily self.

The overall objectives of the present study were to examine the relationship between body ownership and self-orientation perception and to test the hypothesis that body ownership plays a significant role in shaping self-orientation and self-motion perception. We theorized that body ownership should increase the effectiveness of visual self-orientation and self-motion cues because the person’s own body defines the ego-centric spatial reference frame that is central to the interpretation of such cues and for spatial perception in general^21^. To test this prediction, we combined a self-orientation illusion and a full-body ownership illusion in which the ownership of a stranger’s body as seen from the 1PP was manipulated by synchronous (illusion) or asynchronous (control) visual-tactile stimulation. We hypothesized that self-orientation perception would be influenced by body ownership and that the inversion illusion should, therefore, be stronger during synchronous visuo-tactile stimulation than during an asynchronous visuo-tactile condition.

## Methods

### Participants

Thirty-three volunteers participated in the experiment (age = 25.15, SD = 2.98, 21 females). All participants had normal or corrected to normal vision. Participants gave written informed consent before participation and received one cinema ticket as compensation. The experiment was conducted in accordance with the local ethical guidelines, and the experimental procedure was approved by the Regional Ethics Review Board of Stockholm.

### Stimuli and Apparatus

Three-dimensional-image video material of an unknown person sitting in a chair looking down at their legs and feet was prerecorded using two identical cameras placed side by side (CamOne Infinity HD, resolution 1920 × 1080, Touratech AG) and a green-screen setup. The body stimulus (legs) and background (room) were recorded separately. The video material was processed using Finalcut Pro X (Apple Inc., Cupertino, CA). To induce a three-dimensional perception of the visual scene, the pictures obtained using the left and right cameras were placed side-by-side (1920 × 1080). A short demonstration video is available in the supplementary material, showing the video for both the left and right eyes. A change in self-orientation perception was induced using a continuous 7°/sec rotation of the background stimulus around the roll axis (counterclockwise), which further induced self-motion perception. The rotation speed was chosen to be comfortable to the participants and was determined in a pilot study. Two electrodes were attached to the participant’s left index and middle fingers to measure the skin-conductance response (SCR) to a threat using Biopac System MP150 (Goleta, USA).

### Procedure

**Figure 1:**
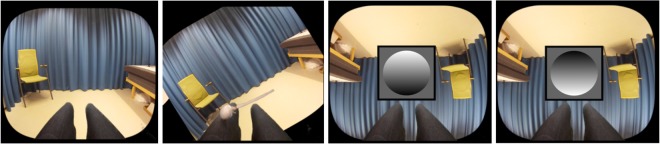
Left figure: Participants saw a stranger’s body form first-person perspective and were exposed to either synchronous or asynchronous visuo-tactile stimulation (within-subject) while the orientation of the visual surroundings presented in the head-mounted display was changing. Right figure: After a 180° rotation, shaded disk stimuli were presented, and the participants were asked to indicate their 3D perception. The image on the left shows a stimulus that is usually perceived as ‘convex’, while that on the right shows a stimulus that is usually perceived as ‘concave’.

During the experiment, participants sat on a chair with their head slightly tilted forward, looking at their legs. Video stimuli were presented using a head-mounted display (HMD, Oculus Rift 2, http://www.oculusvr.com/). The experimental procedure consisted of two different conditions that were presented in a randomized and counterbalanced order: (1) synchronous and (2) asynchronous visuo-tactile stimulations induced through stroking of the participants’ actual legs and the legs seen in the HMDs. The strokes were applied manually, and the experimenter received audio cues to indicate the location and timing of the strokes. Strokes were applied alternatively to both upper legs. The rhythm of the touches followed a semi-regular pattern: one stroke (~1 Hz), a pause of 1 sec, followed by two fast strokes (~0.5 Hz) to one of the legs, then to the other leg (see the video in the supplementary material). The direction of the seen and felt whole-body rotations were the same in both conditions. Each block lasted for 12.5 min, and a total of eight full 360° rotations were presented. The rotation stopped for 17 seconds after a 180° rotation in an upright or upside-down orientation, respectively (see Fig. 1 for illustration). The room upright orientation served as a control condition where we did not expect any reorientation. Participants were presented with three shaded disk stimuli during each such pause in the rotation. Their task was to indicate whether they perceived the disk as convex or concave by pressing one of two buttons with their right index and middle fingers. The shaded disk paradigm was first introduced by Jenkin, Dyde, Jenkin, Howard, and Harris^22^. Assuming that light comes from above, a darker shading on the bottom of the disk induces a convex 3D perception, whereas shading at the top of the disk induces a concave 3D perception^23,24^. A total of 48 disk stimuli were displayed in a random and counterbalanced order in both conditions. No stroking was applied while the disk stimuli were presented. This approach was chosen to avoid distracting participants from the task and to exclude any unspecific effect due to the synchronous or asynchronous stroking. Participants performed a training session prior to the experiment to familiarize themselves with the task.

Illusory ownership of the stranger’s body was measured using questionnaire ratings (subjective measurement) administered after each block, and SCR was induced by threatening the mannequin’s body with a knife (an objective measurement; see further below). Participants filled out a paper questionnaire concerning the sense of ownership (S1), the referral of touch to the stranger’s body (S2, S3), motion perception (S4, S5), orientation perception (S6, S7) and control questions (S8, S9, S10, S11; see Table 1). S1–S3 refers to the original full-body ownership illusion^9^. Statements were rated on a 7-point Likert-scale ranging from −3 (strongly disagree) to +3 (strongly agree), with 0 indicating “neither agree nor disagree”. Participants filled out the questionnaire after each block.

**Table 1:** Results of the Wilcoxon signed-rank test for each statement.

Statement	Median		W	p
	Sync	Async		
S1: I felt as if I was looking at my body	2	1	186.5	0.013
S2: It seemed as if the touch I felt was caused by the stick that touched the body that I saw	2	−2	433	<0.001
S3: The touch I saw was the touch I felt	2	−3	494.5	<0.001
S4: I felt as if I was rotating in the room	3	2	88.5	0.023
S5: I felt as if the room was rotating around the body I saw	−2	1	72	0.215
S6: Sometimes it felt as if I was upside-down compared to gravity	2	1	171	0.049
S6b: How often?	0.500	0.487	308	0.019
S7: Sometimes it felt as if the room was upside-down compared to gravity	1	2	85.5	0.461
S7b: How often?	0.420	0.493	144	0.112
S8: I felt the touch of the stick on my back	−3	−3	10.5	1
S9: I felt as if I had two bodies	−3	−1	46	0.045
S10: I felt as if my body was turning blue	−3	−3	51	0.334
S11: I felt as if the furniture in the room changed shape	−3	−3	35.5	0.782

SCR to a knife threat towards the body stimulus was used as an objective measurement of ownership^9,11,19^. A stabbing knife threat towards the left and right legs of the body stimulus was applied two times during each block (at 4:40 min and 11 min) for a duration of 2 sec each time. Hence, a total of four SCRs were recorded. The video in the supplementary material illustrates the knife threat.

### Data analysis

All data were analyzed using the statistical software package R (R Core Team, 2017). Questionnaire data were analyzed using two-sided Wilcoxon-signed rank tests. Medians and interquartile ranges are reported. SCR data were range-corrected to correct for interindividual variance^25,26^. Each participant’s maximum SCR was determined prior to the start of the first experimental block. The participants were instructed to take a deep breath and then hold it for 2 sec. Each data point was then expressed as a proportion (ratio) of the range of the SCR response according to the following formula: SCR_ratio_ = (SCR_measured_max_ − SCR_measured_min_)/(SCR_max_ − SCR_min_)^26^. SCR magnitude was analyzed using a Wilcoxon-signed rank test, which referred to all SCR responses, including zeros. The relationships between ownership ratings and SCR and ownership ratings and self-motion perception were analyzed using Spearman correlations.

### Shaded disk analysis

Shaded disk data were analyzed using a logistic mixed model approach. The log-odds of saying that a stimulus appeared ‘concave’ was predicted by the stimulus (‘top’ or ‘bottom’ shading), the visuo-tactile stimulation (synchronous, asynchronous) and their interaction. Details about the logistic model are available as supplementary material.

## Results

**Figure 2:**
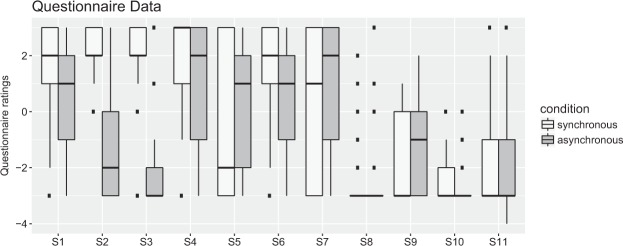
Questionnaire results: S1–S3 capture the illusion statement (‘ownership’ and ‘referral of touch’), S4 reflects ‘self-motion perception’, and S5 reflects ‘perceived room rotation’, S6 measures ‘upside-down orientation perception’, and S7 reflects ‘room upside-down perception’. S8–S11 are control questions. For details about the statements, see Table 1.

**Figure 3:**
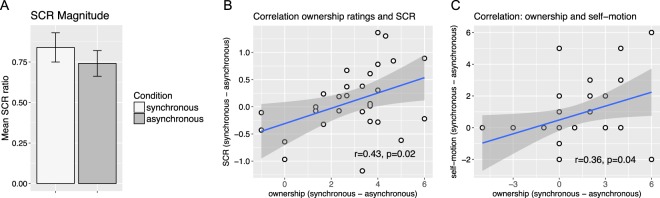
(**A**) Mean skin conductance response (SCR) magnitude in response to a knife threat to the stranger’s body as viewed in the HMDs. SCR magnitude is expressed as the ratio of the value and the maximal SCR registered in each participant (see methods for details). (**B**) Spearman correlation between the difference scores of the ownership rating and the SCR magnitude (ratio): The x-axis reflects the difference between the visuo-tactile synchronous and asynchronous conditions in the ownership statement (S1, Table 1). The y-axis reflects the difference between the visuo-tactile synchronous and asynchronous conditions in terms of SCR magnitude. (**C**) Spearman correlation between subjective body ownership and subjective self-motion: The x- and y-axes reflect the differences between the synchronous and asynchronous conditions in terms of body ownership and self-motion perception ratings, respectively.

The results from the questionnaire are illustrated in Fig. 2. These show that participants experienced stronger ownership during the synchronous (S1: median = 2, IQR = 2) compared to the asynchronous condition (S1: median = 1, IQR = 3) (S1: W = 186.5, p = 0.01). Furthermore, the participants experienced stronger referral of touch to the stranger’s body in the synchronous (S2: median = 2, IQR = 1; S3: median = 2, IQR = 1) than in the asynchronous condition (median = −2, IQR = 3; median = −3, IQR = 1) (S2: W = 433, p < 0.001; S3: W = 494.5, p < 0.001). Interestingly, participants also reported a stronger self-motion illusion (S4: W = 88.5, p = 0.02) and a stronger illusion of being upside-down (S6: W = 171, p = 0.049; S6a: W = 308, p = 0.019) in the synchronous (S4: median = 3, IQR = 2; S6: median = 2, IQR = 2) compared to the asynchronous condition (S4: median = 2, IQR = 4; S6: median = 1, IQR = 3). We note that one of the control statements was more firmly rejected in the asynchronous compared to the synchronous condition (S9: W = 46, p = 0.045), but we will not interpret this further, as the participants clearly denied this (unlikely) experience in both conditions. None of the remaining questionnaire statements showed significant differences between the synchronous and asynchronous conditions. All questionnaire results are presented in Table 1. Interestingly, as illustrated in Fig. 3C, we further observed that the body ownership ratings (synchronous – asynchronous) correlated significantly with the self-motion ratings (r = 0.36, p = 0.04), which suggests that a systematic relationship existed between these two types of percepts, consistent with our hypothesis.

Analysis of the SCR data did not reveal any difference in SCR magnitude to a knife threat between the two visuo-tactile stimulation conditions (see Fig. 3A; Z = −0.71, p = 0.48). Importantly, however, the difference between ownership ratings (synchronous-asynchronous) were significantly correlated with the differences in the threat-evoked SCR magnitude (synchronous-asynchronous) (see Fig. 3B; r = 0.43, p = 0.02). This means that the higher the ownership statement was rated, the stronger the SCR was, which provides objective corroborative evidence that we successfully induced the full-body ownership illusion.

**Figure 4:**
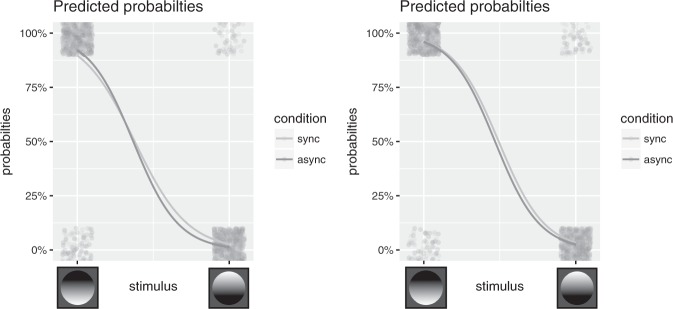
Effect of body ownership on the objective test for self-orientation. The y-axis shows the probability of responding ‘concave’ to a stimulus. The x-axis shows the two possible stimuli: shading on the top (left) and shading on the bottom (right). Left figure: Logistic regression revealed a significant (p < 0.01) interaction between ‘shaded disk stimulus’ and ‘visuo-tactile condition’ when the room was upside-down, indicating a change in perceived orientation in the participants during the full-body ownership illusion. Right figure: No such interaction was found when the room was in an upright condition.

Performance in the shaded disk task during upside-down orientation was analyzed using a logistic mixed-model approach. The analysis revealed that adding the predictor ‘shaded disk stimulus’ (*χ*^2^ = 34.98, df = 1, *p* < 0.001) and adding the interaction between ‘shaded disk stimulus’ and ‘visuo-tactile condition’, which tested our main a priori hypothesis, had a significant influence on performance (*χ*^2^ = 6.24, df = 1, *p* < 0.01) (Fig. 4, Table 2). This means that: (i) stimuli with shading on the top were more likely to be perceived as concave whereas stimuli with shading on the bottom were more likely to be perceived as convex and (ii) synchronous visuo-tactile stimulation modulated the perception of the shaded disc stimulus when the room was presented upside-down. Adding the predictor ‘visuo-tactile condition’ did not improve the model fit significantly (*χ*^2^ = 0.03, df = 1, *p* < 0.86), which suggests that there was no unspecific effect of body ownership on the performance of the shaded disc test. Finally, and as expected, analyzing the performance during upright room orientation revealed a significant effect of ‘shaded disk stimulus’ (*χ*^2^ = 32.59, df = 1, *p* < 0.001); there was no effect of ‘visuo-tactile condition’ (*χ*^2^ = 0.5, df = 1, *p* < 0.48) or the interaction of ‘shaded disk stimulus’ and ‘visuo-tactile condition’ (*χ*^2^ = 0.21, df = 1, *p* < 0.64) (Fig. 4, Table 3). This latter analysis indicates that the shaded disk test worked as expected in the control condition when there was no self-orientation illusion (i.e., in the upright room orientation).

**Table 2:** Results of the logistic mixed model during upside-down orientation including all three predictors.

	Estimate	SE	Z value	p
Intercept	2.16	0.35	6.16	<0.001
Stimulus (convex)	−5.71	0.88	−6.53	<0.001
Condition (asynchronous)	0.32	0.24	1.33	0.18
Interaction	−1.01	0.41	−2.47	0.01

**Table 3:** Results of the logistic mixed model during upright orientation including all three predictors.

	Estimate	SE	Z value	p
Intercept	3.26	0.56	5.8	<0.001
Stimulus (convex)	−6.61	0.96	−6.9	<0.001
Condition (asynchronous)	−0.06	0.29	−0.22	0.83
Interaction	−0.2	0.44	−0.46	0.64

## Discussion

The aim of the present study was to investigate how body ownership contributes to self-orientation perception. To this end, we combined a full-body ownership illusion paradigm with a visually induced reorientation illusion where the visual 3D environment was rotated around the body in view. Subjective questionnaire ratings indicated that the feelings of self-motion (S4) and self-orientation (S6) were modulated by body ownership because these sensations were stronger in the synchronous visuo-tactile condition than in the asynchronous condition. Moreover, the higher the ownership ratings, the stronger the rating of perceived self-motion. Interestingly, the questionnaire findings were supported by objective measurements of self-orientation perception using the shape-from-shading task. This behavioral paradigm showed that perception of the shaded disk stimuli was affected by illusory body ownership such that the full-body ownership illusion changed participants perceived self-orientation. In summary, these novel findings suggest that the sense of body ownership determines perceived self-orientation as well as self-motion perception, a conclusion that has an important bearing on fundamental theories of how the sense of self and space are linked in the human mind.

Previous studies have shown that strong visual cues induce a feeling of self-motion and reorientation^27,28^. This well-known phenomenon was clearly observed in our questionnaire data, where subjects reported moderately strong self-motion perceptions as well as a change in perceived orientation, even in the asynchronous control condition (S4 and S6, see Table 1). To the best of our knowledge, no study has investigated whether body ownership influences self-motion and orientation perception over and above the effects of visual cues. Our results show that body ownership augments these spatial orientation sensations, which suggests that the multisensory interactions that underpin the sense of body ownership also shape self-orientation perception. What could be the mechanism behind this functional interaction? One parsimonious explanation is that the synchronous visual-tactile information that elicited the feeling of ownership of the body in view made the visual information from the surrounding environment more potent as a self-motion cue (“The scene is rotating around my body, not just around another person’s body”), resulting in a stronger weighting of visual information, which in turn resulted in boosted feelings of self-motion and self-reorientation. In contrast, in the asynchronous condition, body ownership was reduced, which made the visual cues less informative as self-motion cues; therefore, vision was weighted less than the vestibular information, leading to a reduction in perceived self-motion and reorientation.

This interpretation, that body ownership influences the interpretation of visual self-motion and self-orientation cues, is consistent with the findings of previous studies showing that body ownership influences visual perceptual processes such as visual size^29–31^ and visual distance perception^31^, as well as visual awareness^32^. From this “embodied vision” perspective, the present results constitute a new example of body ownership modulating the 3D perception of shaded disk stimuli, which is an indicator of a change in self-orientation perception. However, an important difference from the above studies is that in the present study, we demonstrate an effect of body ownership on the perceived spatial orientation of oneself in the field of gravity. This is fundamentally different from ownership-induced changes in visuospatial object-size and distance perception^29–31^, although the interaction between the representation of one’s own physical self and the external space is a common denominator.

In this study, several limitations are possible. The stroking was absent during the 17-second periods when the participants performed the shaded disk task. It is possible that this could have reduced the difference in illusion strength between the synchronous and asynchronous illusion conditions, which in turn would have worked against the hypothesis that we were testing, leading to reduced effect sizes. However, our key effects on self-orientation were statistically significant. It should also be noted that full-body ownership illusions can be maintained for periods longer than 17 seconds without dynamic visuo-tactile stimulation after first having been elicited in the usual way with synchronous visuo-tactile stimulation^33^. Another possible concern is that the upside-down visual presentation of the room could have reduced the body ownership illusion compared to the normal upright presentation. However, informal pilot experiments indicated that the full-body ownership illusion was robust across the different room orientations, and we also know from previous experiments that the illusion works very well in different spatial environmental contexts^30,31,33,34^; again, any such effect would have worked against the hypothesis that we were testing with the shaded disk paradigm.

To conclude, the present study provides evidence that the sense of body ownership influences self-orientation perception. Thus, the way we perceive the world from an egocentric perspective and our sense of self-orientation is determined by the multisensory perception of body ownership. Future studies should aim to investigate the underlying neural mechanisms that contribute to the coherent experience of our bodily self and its perceived location in space.

## Electronic supplementary material

Demo Video

Supplementary Material
