# Supplementary material for: Body ownership shapes self-orientation perception
Source: Sci Rep. 2018 Oct 30;8:16062. doi: 10.1038/s41598-018-34260-7 (PMC6207657; doi:10.1038/s41598-018-34260-7)
Supplement: Supplementary file 2 — Supplementary Material [file 41598_2018_34260_MOESM2_ESM.docx]

Body ownership shapes self-orientation perception

Nora Preuss, B. Laufey Brynjarsdottir, H. Henrik Ehrsson

Department of Neuroscience, Karolinska Institutet, Stockholm, Sweden

Corresponding author:

Nora Preuss

Tomtebodavägen 16

17165 Solna

Sweden

Email: [nora.preuss@ki.se](mailto:nora.preuss@ki.se)

phone: (+46) 8 524 87 066

*Supplementary material of shaded disk analysis*

Shaded disk data were analyzed using a logistic mixed model approach using the statistical program R^1^. The log-odds of saying that a stimulus appeared ‘concave’ was predicted by the stimulus (‘top’ or ‘bottom’ shading), the visuo-tactile stimulation (synchronous, asynchronous) and their interaction. The model included a fixed intercept γ_00_, a fixed effect for stimulus γ_10_, a fixed effect for stimulation condition γ_20_ and a fixed effect for the interaction γ_30_. In addition, υ_0i_ and υ_1i_ were the random effects of the intercept and the predictor stimulus for each participant *i.* A random effect for ‘stimulus’ was included for two reasons: (1) A pilot experiment identified qualitative differences between the two stimuli. The top-shaded stimulus (‘concave’) turned out to be less salient in a pilot test; the perception of the ‘concave disks’ (with shading on top) depended more on the assumed direction of light, whereas the perception of a ‘convex disk’ (with shading on bottom) was more stable^2, 3^. (2) Including a random effect for ‘stimulus’ significantly improved the model fit. Performance for stimuli with bottom shading during synchronous visuo-tactile stimulation served as a reference category. The log-linear regression model was

(1)

$$\ln\left( \frac{p\left( concave \right)}{(1-p\left( concave \right))} \right)=b_{oi}+{Stimulus*b}_{1i}+{Stimulation* b}_{2i}+Stimulation*Stimulus*b_{3i}$$

(2)

$$b_{0i}=\gamma_{00}+\upsilon_{0i}$$

$$b_{1i}=\gamma_{10}+\upsilon_{1i}$$

$$b_{2i}=\gamma_{20}$$

$$b_{3i}=\gamma_{30}$$

References

[1] R Core Team. *R: A language and environment for statistical computing.* Vienna, Austria, R Foundation for Statistical Computing (2017).

[2] Kleffner, D. A., & Ramachandran, V. S.. On the perception of shape from shading. *Attention, Perception, & Psychophysics, 52*(1), 18-36 (1992).

[3] Ramachandran, V. S.. Perception of shape from shading. *Nature, 331*(6152), 163-166 (1988).
